# A prognostic model for breast cancer survival based on PCD and m6A gene interactions

**DOI:** 10.3389/fimmu.2025.1711910

**Published:** 2025-11-20

**Authors:** Weimiao Li, Haocheng Bai, Jiajing Yang, Meng Zhan, Shuqun Zhang, Guoxu Zheng

**Affiliations:** 1 The Comprehensive Breast Care Center, The Second Affiliated Hospital of Xi’an Jiaotong University, Xi’an, China; 2 State Key Laboratory of Holistic Integrative Management of Gastrointestinal Cancers and Department of Immunology, Fourth Military Medical University, Xi’an, China

**Keywords:** breast cancer, programmed cell death, N6-methyladenosine, immune evasion, prognostic genes

## Abstract

**Introduction:**

Breast cancer (BC) is the most prevalent malignancy in women, with patient outcomes heavily influenced by complex molecular mechanisms like programmed cell death (PCD) and RNA methylation. While some studies have investigated how specific PCD types and N6-methyladenosine-related genes (m6A-RGs) are associated with breast cancer, the research on combined PCD mechanisms and their role in breast cancer development is limited. This study integrates PCD-related genes (PCD-RGs) and m6A-RGs to offer new insights for breast cancer clinical treatment.

**Methods:**

Transcriptomic data and related genes were respectively retrieved from public databases and published literature. First, PCD-m6A genes identified through the correlation scoring and differentially expressed genes were intersected to obtain candidate genes. Furthermore, to infer potential causal relationships between gene expression and survival, we applied a two-sample Mendelian randomization approach using summary-level data from public databases. Therefore, prognostic genes were further obtained through Mendelian randomization and regression analyses, and a prognostic model was then constructed. Additionally, functional enrichment, immune infiltration, and drug sensitivity analyses were conducted. Finally, the expression intensity of prognostic genes was verified by RT-qPCR and IHC.

**Results:**

Through a series of analyses, seven prognostic genes were identified. Following this, the prognostic model has been demonstrated to have a certain degree of accuracy as indicated by both transcriptomic public sets. Successively, enrichment analysis revealed numerous pathways, among which herpes simplex virus 1 infection was notable; its relevance lies in overlapping immune evasion pathways with BC, a core focus of our investigation. Immune cell infiltration analysis revealed that 11 immune cell types, including M1 macrophages, exhibited significant differences between high and low groups. A key finding from drug sensitivity analysis was that the high-risk group exhibited significantly increased sensitivity to several drugs, including CCT018159, rapamycin, vinblastine, metformin, and roscovitine. The expression levels of MYD88, DAXX and ANXA5 were significantly upregulated in the control samples compared to breast cancer samples. Moreover, the expression levels of SESN3, CRIP1, DPP4 and PIK3CA were significantly upregulated in breast cancer samples compared to control samples.

**Discussion:**

This study constructed a risk model based on seven prognostic genes, offering new potential strategies for breast cancer therapy.

## Graphical Abstract

Flow chart of this study.

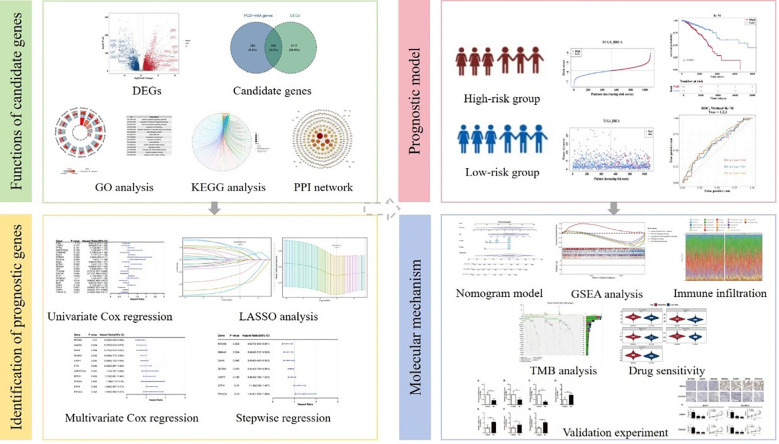

## 1 Introduction

Breast cancer (BC) is the most prevalent malignant tumor among women worldwide, posing a severe threat to female life and health (1). In 2020, approximately 2.3 million new cases and 685,000 deaths were reported. This accounted for over 24% of global cancer cases among females and about 15% of cancer-related deaths, making it the second leading cause of cancer mortality (2). Based on the expression status of estrogen receptor, progesterone receptor, and human epidermal growth factor receptor 2 (HER2), BC is typically classified into four subtypes: Luminal A, Luminal B, HER2-enriched, and triple-negative breast cancer (3). This disease exhibits significant heterogeneity. Advances in surgery, chemotherapy, radiotherapy, targeted therapy, and endocrine therapy have made progress (4), but BC remains associated with substantial morbidity and mortality. While early diagnosis via mammography screening and combined diagnostics has significantly enhanced overall survival and prognosis for early-stage patients with breast cancer (5), predicting outcomes for advanced and metastatic breast cancer remains challenging. This difficulty arises from the limited accuracy of known clinical, pathological, and molecular features. In recent years, immunotherapy has emerged as a promising avenue for cancer treatment, which harnesses the body’s immune system to target and eradicate tumor cells (6). Although several prognostic indicators for breast cancer currently exist, they still exhibit significant limitations in accurately predicting patient survival outcomes, such as risks of recurrence and variations in treatment response. These shortcomings hinder their ability to meet the clinical demands of personalized treatment decision-making. Thus, there is an urgent need to explore novel prognostic biomarkers to address this gap.

Programmed cell death (PCD) is an intrinsic property of all cellular life forms (7). PCD primarily includes pyroptosis, apoptosis, necroptosis, ferroptosis, cuproptosis, and PANoptosis. Recent studies indicate that PCD participates in various pathophysiological processes. It is crucial for host defense against pathogens and organismal development. This mechanism serves as a key mediator in the pathogenesis of diseases such as autoimmune disorders, cancer, neurodegenerative diseases, immunodeficiency, and developmental abnormalities (8, 9). Moreover, PCD is closely associated with innate immunity and plays a pivotal role in regulating the immunosuppressive tumor microenvironment (10, 11). Research on the various types of PCD in breast cancer pathogenesis has been conducted, including studies on molecular clustering and prognostic signatures related to PANoptosis (12), as well as prognostic models that involve cuproptosis/ferroptosis-related genes (13). However, there is still limited research that integrates PCD mechanisms with breast cancer development. While existing studies have acknowledged the role of PCD in cancer, research focusing on PCD-related genes (PCD-RGs) in BC remains largely confined to basic mechanistic investigations. Their translational relevance in clinical prognostic assessment has yet to be fully elucidated, and functional validation coupled with clinical translation in this context remains notably scarce, warranting further in-depth investigation.

Epigenetics connects environmental influences and genetic factors, referring to heritable modifications that regulate gene expression without changing the nucleotide sequences. Over 160 types of RNA modifications exist in organisms (14). Among these, N6-methyladenosine (m6A) is the most prevalent internal methylation modification at the N6 position of adenosine (15). Eukaryotic biological processes are regulated by m6A through “writers”, “readers”, and “erasers”, influencing the onset and progression of multiple diseases. Current m6A research focuses largely on tumors, with established roles in lung, cervical, and other cancers (15, 16). In breast cancer, m6A-related genes have been investigated for prognostic prediction and immune characterization (17). Over the past few years, accumulating evidence has demonstrated that m6A can regulate gene expression, thereby influencing multiple PCD processes (18, 19). Studies have investigated the potential functional implications of m6A regulation in PANoptosis among patients with bladder cancer (20). Although m6A, as a pivotal mechanism in RNA regulation, has been demonstrated to be associated with breast cancer progression, current research predominantly focuses on its individual effects. Investigations that integrate m6A with other core molecular mechanisms, such as PCD, to analyze their coordinated regulation in BC pathogenesis remain notably limited, thereby failing to elucidate the comprehensive landscape of the intricate regulatory network in BC.

To thoroughly investigate the mechanisms linking programmed cell death and m6A in BC, it is essential to identify prognostic genes with a causal relationship to the disease. Mendelian randomization (MR) uses genetic variants as instrumental variables. These variants are determined at birth and remain stable, unaffected by environmental factors (21). By leveraging instrumental variables to link exposures and outcomes, MR mitigates confounding bias, enhancing reliability and accuracy in causal inference beyond traditional epidemiological limitations (22). Current research on breast cancer-associated genes predominantly relies on correlational analyses. While this approach can identify genes with statistical associations, it fails to effectively distinguish between ‘causal relationships’ and ‘non-causal accompaniments’, thereby limiting the clinical translational potential of candidate functional genes. In contrast, studies employing MR to establish causal links between genes and BC are still in their nascent stage, with both relevant data and validation evidence remaining notably scarce.

This study aims to develop and validate a prognostic model for breast cancer by integrating bioinformatics and mendelian randomization methods, based on the interactions of m6A-RGs and PCD-RGs. Next, the relevant mechanisms of prognostic genes were explored through gene set enrichment analysis, immune infiltration, drug sensitivity analysis, which offered new insights for the clinical management of breast cancer.

## 2 Materials and methods

### 2.1 Data collection

The TCGA-BRCA dataset was retrieved from TCGA (https://portal.gdc.cancer.gov/) on May 31, 2024, for the training cohort. It encompassed 1,104 breast cancer (BC) tissue samples and 113 control tissue samples, containing 1082 breast cancer samples with survival information (23). TCGA-BRCA was denoted as TCGA-BC.

Breast cancer-related transcriptome datasets were collected from the Gene Expression Omnibus database (https://www.ncbi.nlm.nih.gov/geo/) in the validation cohort. The GSE42568 (GPL570 platform) consisted of 104 breast cancer tissue samples and 17 control samples (containing 104 breast cancer samples with survival information) (24).

Additionally, 27 N6-methyladenosine-related genes (m6A-RGs) were identified in published literature (**Supplementary Table 1**) (25).

Additionally, 1,548 programmed cell death related genes (PCD-RGs) were reported in the literature (**Supplementary Table 2**) (26).

The genome-wide association studies (GWAS) data of expression Quantitative Trait Loci for candidate genes were retrieved from the Integrative Epidemiology Unit (IEU) Open GWAS database (https://gwas.mrcieu.ac.uk/). The breast cancer dataset was obtained from the IEU Open GWAS database by searching with the keyword “breast cancer”. The dataset identified was ukb-b-16890, including a total of 9,851,867 single-nucleotide polymorphisms (SNPs) derived from 462,933 European samples (breast cancer: 10,303, control: 452,630).

### 2.2 Differential expression analysis

Differential expression analysis was used to identify differentially expressed genes between BC and control groups in TCGA-BC via the DESeq2 package (version 1.42.0) (27) (|log_2_Fold Change (FC)| > 0.5 and *p* < 0.05).

### 2.3 Identification and functions of candidate genes

The Spearman method was employed to analyze the correlation between m6A-RGs and PCD-RGs. Genes that were significantly correlated with PCD-m6A genes were selected (|r| > 0.3 and *p* < 0.05). Following this, DEGs and PCD-m6A genes were intersected to identify candidate genes. Specifically, to explore the biological pathways involving the candidate genes, a functional enrichment analysis was conducted of the candidate genes using the clusterProfiler package (version 4.7.1.003) (28), drawing data from the Kyoto encyclopedia of genes and genomes (KEGG) and gene ontology (GO) databases (*p* < 0.05). Moreover, The STRING (https://string-db.org) was employed to build the protein-protein-interaction network to explore the interaction relationships of candidate genes at the protein level (confidence = 0.9).

### 2.4 Mendelian randomization analysis

Based on Mendelian randomization method, candidate genes that had a causal relationship with breast cancer were identified via TwoSampleMR package (version 0.6.4) (29). Candidate genes were considered as predictors, with breast cancer identified as the outcome of interest.

Initially, SNPs were selected with *p* < 5 × 10^–8^, clump = TRUE, R^2^ < 0.001, kb > 100, and SNPs > 3. Instrumental variables were calculated to be F-statistic, with F > 10. Moreover, the directionality of exposure factors was tested. After that, MR combined five algorithms for MR analysis, including MR-Egger (30), inverse variance weighted (IVW) (31), weighted median (32), weighted mode (33), and simple mode (29). The IVW method served as the primary measure for determining statistical significance (*p* < 0.05). In turn, correlation analysis was conducted with a scatter plot, forest plot, and randomness analysis with a funnel plot. Moreover, the robustness of Mendelian randomization analysis results was assessed via a sensitivity analysis, comprising heterogeneity test (*p* > 0.05) (34), horizontal pleiotropy test (*p* > 0.05) (35), and Leave-One-Out analysis. Additionally, the Steiger directional test was applied to eliminate the prospect of reverse causation (result = TRUE, *p* < 0.05) (36).

Ultimately, the candidate genes specifically linked to breast cancer were obtained through Mendelian randomization analysis and documented as key genes. These key genes were used for further analysis.

### 2.5 Identification of prognostic genes

In order to further screen out the prognostic genes related to the prognosis of breast cancer from the key genes, various regression analysis methods were employed. Initially, based on breast cancer tissue samples from TCGA-BC, the survival package (version 3.5.3) (37) was employed to construct a univariate Cox regression analysis (hazard ratio ≠ 1, *p* < 0.2) with proportional hazards (PH) assumption test (*p* > 0.05) to identify survival-associated genes. Subsequently, the glmnet package (version 4.1.4) was utilized to perform least absolute selection and shrinkage operator (LASSO) (log(lambda.min) ≠ 0). The genes identified through LASSO regression were initially evaluated for the PH assumption (*p* > 0.05), followed by multivariate Cox analysis. Afterwards, the model was screened and validated by stepwise regression analysis (*p* < 0.05). Finally, the key genes identified through all the aforementioned analyses were defined as prognostic genes which were utilized to construct risk model.

### 2.6 Construction and validation of risk model

Based on prognostic genes, the TCGA-BC dataset was utilized as a training cohort (n = 1,082) to construct a risk model, while the GSE42568 dataset acted as a validation cohort (n = 104) to validate the risk model for predicting outcomes in patients with breast cancer. Firstly, in TCGA-BC, based on the relative expression intensity of prognostic genes and regression coefficients, risk scores were computed for patients with BC. The formula used was $\text{Riskscore}=\sum_{\text{i}=1}^{\text{n}}\text{coef}({\text{gene}}_{\text{i}})*\text{expr}({\text{gene}}_{\text{i}})$, where expr signified the expression level of prognostic genes and coef denoted the coefficient associated with prognostic genes. Breast cancer specimens were binned into two risk groups via an optimal cutoff value. A risk curve scatter plot was plotted, and the expression intensity of prognostic genes was displayed. Furthermore, the Kaplan-Meier survival curve for overall survival of the two risk groups was generated using the survminer package (version 0.4.9) (38) (*p* < 0.05). Finally, the survivalROC package (version 1.0.3) (39) was leveraged to generate a receiver operating characteristic (ROC) curve for assessing 1, 2, and 3 years survival prospects. Moreover, the risk model underwent validation in a validation cohort.

Additionally, to explore the prognostic value of prognostic genes, in the BC samples with survival information of TCGA-BC, based on the optimal cut-off value of prognostic gene expression, BC samples were divided into high-expression and low-expression groups. Then, the Kaplan-Meier survival curves for overall survival of patients in the high and low expression groups were analyzed using the survminer package (version 0.4.9), and the log-rank test was applied to compare the survival differences between the two groups (*p* < 0.05).

### 2.7 Independent prognostic analysis

To explore the independent factors related to prognosis, an independent prognostic analysis was conducted by integrating risk scores, age, gender, T.stage, N.stage, and M.stage. Cox regression analysis was utilized to identify an independent prognostic factor paired with breast cancer (*p* < 0.05). Additionally, based on independent prognostic factors, the rms package (version 6.5-1) (40) was utilized to construct a nomogram model to explore the diagnostic value of independent prognostic factors. Notably, 1-, 2-, and 3-year calibration curves and ROC curve were utilized to verify the accuracy of the nomogram.

### 2.8 Correlation of risk scores with clinical characteristics

The relationship of risk score to the clinical characteristics of BC was evaluated via a series of analyses. First, the distinctions in risk score among different clinical characteristics were compared. The survival package was employed to construct Kaplan-Meier curves, which assessed distinctions in overall survival between two risk groups under various subgroups of clinical characteristics (*p* < 0.05).

### 2.9 Gene set enrichment analysis

To scrutinize the intrinsic mechanisms associated with prognostic genes, GSEA was carried out. Initially, the DESeq2 package was employed to differentially analyze the two risk groups and sort the log_2_FC from largest to smallest. Furthermore, GSEA was conducted using the gseKEGG function from the clusterProfiler package to explore the functional pathways associated with the prognostic genes. A normalized enrichment score (|NES|) > 1 and *p* < 0.05 were considered significant. The top 5 pathways were visualized.

### 2.10 Analysis of immune cell infiltration

The abundance of immune cell infiltration between the high-risk and low-risk groups was also investigated. CIBERSORT algorithm (version 1.03) (41) was employed to assess enrichment of 22 immune cell types in TCGA-BC. Afterwards, deviations in enrichment of immune cells were analyzed (*p* < 0.05). Additionally, the relationships among prognostic genes and various immune cells, the interactions between different immune cells, and the connections among prognostic genes within the TCGA-BC were analyzed using the psych package.

### 2.11 Mutation status of patients with breast cancer in two risk groups

To better understand variations in driver genes between two risk groups, the maftools package (version 2.14.0) (42) was utilized to analyze gene mutations in two risk groups and display the top 20 high-frequency mutated genes in a tumor mutational burden waterfall plot.

### 2.12 Drug sensitivity analysis

Additionally, the sensitivity of the high-risk and low-risk groups to the drug was also analyzed. The information on chemical drugs for breast cancer and their half maximal inhibitory concentration (IC_50_) was retrieved from the Genomics of Drug Sensitivity in Cancer databases (http://cancerrxgene.org). The pRRophetic package (version 0.5) (43) was utilized to calculate IC_50_ values of drugs in patients with breast cancer samples in TCGA-BC. Subsequently, the psych package was used for Spearman’s correlation analysis of drug IC_50_ values versus risk scores (cor) > 0.3 and *p* < 0.05). The top five drugs were presented according to their adjusted *p*-value.

### 2.13 Reverse transcription quantitative polymerase chain reaction

To verify the expression levels of the prognostic genes in the samples, RT-qPCR was conducted. RNA specimens from 32 matched pairs of breast cancer and adjacent paracancerous tissues were obtained from Xi’an Jiaotong University Second Affiliated Hospital. The inclusion criteria encompassed sufficient bone marrow, hepatic, and renal function, a minimum expected survival of three months, and provision of informed consent. The exclusion criteria comprised incomplete case information, mortality due to postoperative complications, and the presence of concurrent malignancies. This study complied with the Declaration of Helsinki (2013 revision) and was formally approved by the hospital’s Ethics Committee (Approval No. 2024YS060). All participants provided written informed consent. In the RT-qPCR, total RNA was extracted using manufacturer-specified protocols. The mRNA underwent reverse transcription with the SweScript First Strand cDNA Synthesis Kit, followed by quantitative PCR amplification using SYBR Green qPCR Master Mix. Primer sequences are detailed in **Supplementary Table 3**. Moreover, mRNA expression levels were normalized to GAPDH and quantified via the 2^−ΔΔCt^ method. Statistical significance (*p*-values) was determined using GraphPad Prism (version 6).

### 2.14 Immunohistochemistry

Fresh tumor specimens were fixed in neutral buffered formalin for a duration of 24 hours at ambient temperature. Subsequently, the samples underwent embedding and processing in accordance with established protocols. Tissue sections were deparaffinized utilizing a series of graded ethanol solutions and subsequently rehydrated. Antigen retrieval was conducted for 30 minutes using an antigen retrieval solution. Following this, the sections were stained with PIK3CA, SESN3, ANXA5, MYD88, DPP4, DAXX, and CRIP1.

### 2.15 siRNA transfection

BRCA cells were seeded into 6-well plates at a density ranging from 50,000 to 100,000 cells per well and cultured overnight to achieve 50–70% confluency. Each small interfering RNA (siRNA) (siPIK3CA#1: GGAUCAGAUGAAUUCACUATT; siPIK3CA#2: CCACAAAUUAUCAUAGAAUTT; siCRIP1#1:GUGAUUCUGUGCUACUAUTT; siCRIP1#2:GGAACAAGUGCUUGGUCAUTT) was diluted to a working concentration of 20 μM in nuclease-free water. For the transfection process, 5 μL of Lipofectamine 2000 was combined with 100 μL of Opti-MEM I medium and incubated for 5 minutes at room temperature. Subsequently, 50 nM of each siRNA was added to the mixture, resulting in a total volume of 200 μL. Following a 20-minute incubation at room temperature, the siRNA-lipid complex was added dropwise to each well containing cells in Opti-MEM I medium. The cells were then incubated at 37 °C with 5% CO2 for 48 hours to facilitate PIK3CA or CRIP1 knockdown. Following transfection, the cells were subjected to RNA extraction to verify the efficiency of the knockdown.

### 2.16 CCK8 assay

The proliferation of PIK3CA or CRIP1 knockdown breast cell lines was evaluated utilizing the CCK8 assay. Cells were seeded in 96-well plates at a density of 1,000 cells per well, with three replicates for each condition. The experimental setup included continuous treatment with 0.5 μM of the drug or no treatment, alongside blank wells as controls. Proliferation assessments were conducted every 24 hours over a 3-day period. At each specified time point, 10 μL of CCK8 reagent was added to each well, followed by a 3-hour incubation at 37 °C. Absorbance was subsequently measured at 450 nm using a microplate reader. Proliferation curves were generated and analyzed using GraphPad Prism 6 software.

### 2.17 Statistical analysis

All data were managed utilizing R language software (version 4.2.2). The Wilcoxon test was employed for analytical comparisons, with *p* < 0.05 considered statistically significant.

## 3 Results

### 3.1 The functions of 455 candidate genes related to m6A and PCD in breast cancer

**Figure 1:**
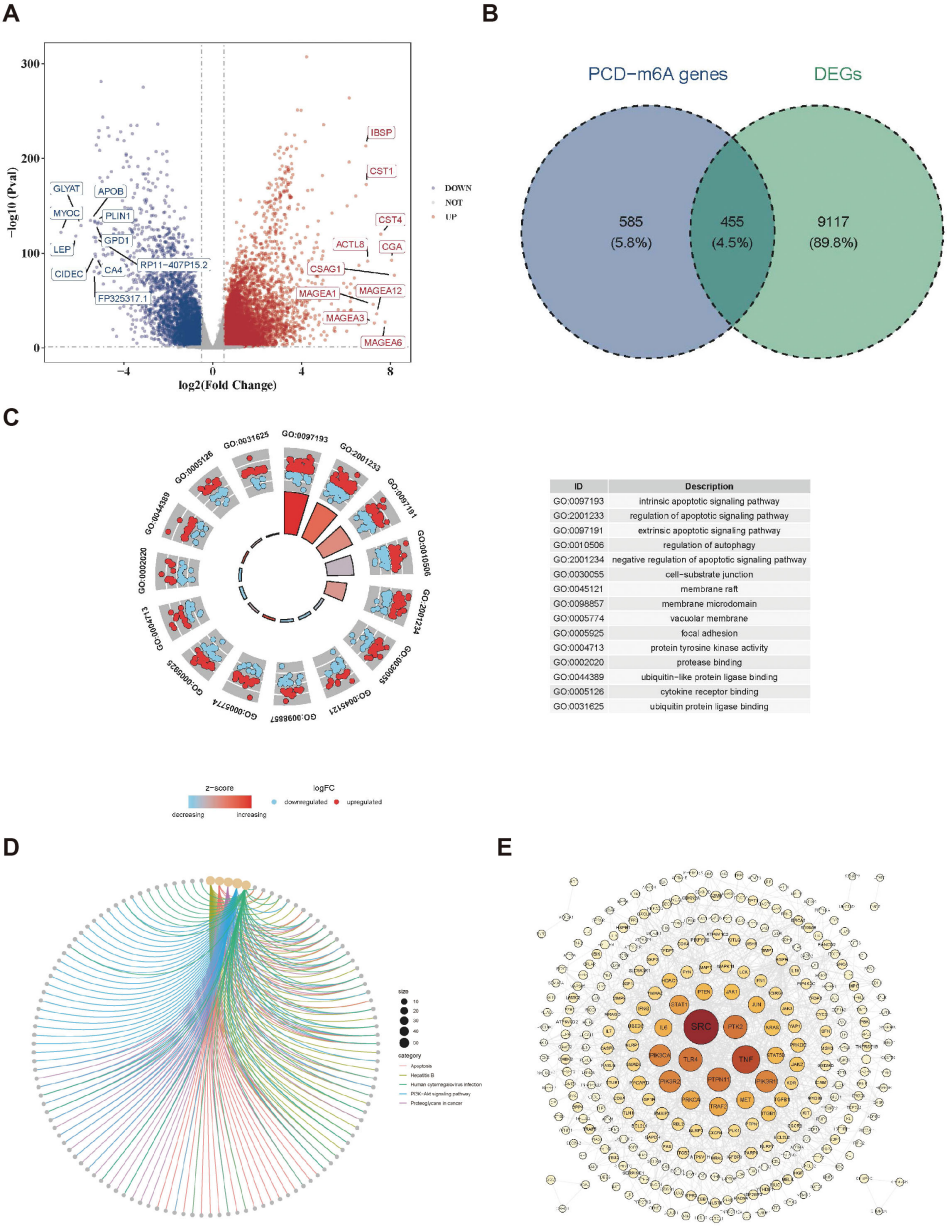
Identification of candidate genes. **(A)** Volcano plot of differentially expressed genes in the BC dataset. **(B)** Venn map acquisition of 455 intersection genes of differentially expressed genes and PCD-m6A-RGs. **(C)** Gene Ontology (GO) enrichment analysis of 455 candidate genes. **(D)** Kyoto encyclopedia of genes and genomes (KEGG) pathway enrichment analysis of 455 candidate genes. **(E)** Construction of a PPI network for 455 candidate genes.

By differential expression analysis, 9,572 differentially expressed genes, with 5,933 upregulated and 3,639 downregulated in the BC group were obtained (**Figure 1A**). Meanwhile, 1040 PCD-m6A genes were obtained from Spearman correlation analysis between 27 m6A-RGs and 1,548 PCD-RGs (|r| > 0.3 and *p* < 0.05). By analyzing the overlap between 9,572 differentially expressed genes and 1,040 PCD-m6A genes, 455 candidate genes were obtained (**Figure 1B**). Notably, GO analysis showed that 455 candidate genes were significantly enriched in 2,705 biological process entries, such as the intrinsic apoptotic signaling pathway. Additionally, there were 200 entries related to cellular components, such as cell-substrate junctions, and 234 entries related to molecular functions, including protease binding (**Figure 1C**, **Supplementary Table 4**). Furthermore, 160 KEGG pathways were identified, such as Hepatitis B and apoptosis (**Figure 1D**, **Supplementary Table 5**). At the protein level, there were interactions among 309 candidate genes (**Figure 1E**), with SRC and TNF exhibiting a higher number of connections and interactions with other proteins.

### 3.2 Acquisition of 54 key genes among candidate genes

A two-sample MR analysis involved 455 candidate genes as exposure variables, with breast cancer as the outcome. About 54 genes were identified and were used as key genes (*p* < 0.05). Consequently, 26 protective factors were identified (OR < 1, *p* < 0.05), while 28 risk factors were also identified (OR > 1, *p* < 0.05) (**Table 1**). Analyzing the correlation between exposure factors and outcomes using a scatter plot revealed that 28 genes were positively correlated, while 26 genes were negatively correlated. Forest plots indicated that the effect value of 26 genes was less than 0, while the effect values of the 28 risk genes were greater than 0. SNP numbers were largely symmetrical on both sides of the line and corresponding to Mendel’s second law. Moreover, sensitivity analyses revealed that all 54 exposure factors displayed no evidence of heterogeneity (*p* > 0.05) (**Supplementary Table 6**). Additionally, there was no indication of horizontal pleiotropy between the 54 exposure factors and the outcome (*p* > 0.05) (**Supplementary Table 7**). In Leave-One-Out, no significant bias was observed, supporting the reliability of the results. Finally, when using breast cancer as the exposure factor and the 54 genes as the outcome in the Steiger test for reverse causality, the directional relationship was determined to be “TRUE”. There was no reverse causality between the 54 exposure factors and the outcome (*p* < 0.05) (**Supplementary Table 8**). Overall, 54 genes were recorded as key genes.

**Table 1:** Results of Mendelian randomization analysis.

Exposure	Method	Nsnp	B	Se	Pval	Or	Or_lci95	Or_uci95	Symbol	Ensembl
eqtl-a-ENSG00000172403	Inverse variance weighted (fixed effects)	7	0.001933	0.000983	0.049306	1.001935	1.000006	1.003867	SYNPO2	ENSG00000172403
eqtl-a-ENSG00000003400	Inverse variance weighted (multiplicative random effects)	7	-0.00398	0.001418	0.00505	0.996031	0.993266	0.998804	CASP10	ENSG00000003400
eqtl-a-ENSG00000164111	Inverse variance weighted (fixed effects)	12	-0.00077	0.000385	0.045947	0.999231	0.998477	0.999986	ANXA5	ENSG00000164111
eqtl-a-ENSG00000175793	Inverse variance weighted (fixed effects)	3	-0.00295	0.001467	0.044255	0.997052	0.994189	0.999924	SFN	ENSG00000175793
eqtl-a-ENSG00000164938	Inverse variance weighted (fixed effects)	8	0.001324	0.000653	0.042711	1.001325	1.000043	1.002608	TP53INP1	ENSG00000164938
eqtl-a-ENSG00000169230	Inverse variance weighted (fixed effects)	9	-0.00081	0.000395	0.039589	0.999187	0.998412	0.999961	PRELID1	ENSG00000169230
eqtl-a-ENSG00000143799	Inverse variance weighted (fixed effects)	16	0.00098	0.00047	0.037137	1.000981	1.000058	1.001904	PARP1	ENSG00000143799
eqtl-a-ENSG00000065361	Inverse variance weighted (fixed effects)	4	-0.00175	0.000839	0.03692	0.998252	0.996612	0.999894	ERBB3	ENSG00000065361
eqtl-a-ENSG00000002330	Inverse variance weighted (fixed effects)	3	0.00388	0.001853	0.036275	1.003888	1.000248	1.00754	BAD	ENSG00000002330
eqtl-a-ENSG00000153162	Inverse variance weighted (fixed effects)	13	0.001599	0.000762	0.035979	1.0016	1.000105	1.003098	BMP6	ENSG00000153162
eqtl-a-ENSG00000065534	Inverse variance weighted (fixed effects)	13	0.001765	0.000841	0.035779	1.001766	1.000117	1.003418	MYLK	ENSG00000065534
eqtl-a-ENSG00000169429	Inverse variance weighted (fixed effects)	12	0.001188	0.000558	0.033236	1.001189	1.000094	1.002285	CXCL8	ENSG00000169429
eqtl-a-ENSG00000091592	Inverse variance weighted (fixed effects)	10	-0.00118	0.000547	0.031487	0.998825	0.997756	0.999896	NLRP1	ENSG00000091592
eqtl-a-ENSG00000196296	Inverse variance weighted (fixed effects)	5	-0.00249	0.001154	0.030944	0.997514	0.995261	0.999772	ATP2A1	ENSG00000196296
eqtl-a-ENSG00000084073	Inverse variance weighted (fixed effects)	5	0.00221	0.001021	0.030375	1.002212	1.000209	1.004219	ZMPSTE24	ENSG00000084073
eqtl-a-ENSG00000072274	Inverse variance weighted (fixed effects)	9	-0.00134	0.000614	0.029437	0.998663	0.997461	0.999866	TFRC	ENSG00000072274
eqtl-a-ENSG00000104549	Inverse variance weighted (fixed effects)	6	0.002119	0.000969	0.028727	1.002121	1.00022	1.004025	SQLE	ENSG00000104549
eqtl-a-ENSG00000164081	Inverse variance weighted (fixed effects)	11	0.001892	0.000851	0.026293	1.001894	1.000223	1.003567	TEX264	ENSG00000164081
eqtl-a-ENSG00000102606	Inverse variance weighted (fixed effects)	11	0.000982	0.000433	0.023456	1.000983	1.000133	1.001834	ARHGEF7	ENSG00000102606
eqtl-a-ENSG00000127415	Inverse variance weighted (fixed effects)	6	0.001614	0.000712	0.02343	1.001615	1.000218	1.003014	IDUA	ENSG00000127415
eqtl-a-ENSG00000137831	Inverse variance weighted (fixed effects)	5	-0.00192	0.000848	0.023428	0.99808	0.996422	0.99974	UACA	ENSG00000137831
eqtl-a-ENSG00000172936	Inverse variance weighted (fixed effects)	6	-0.00293	0.001286	0.022873	0.997078	0.994568	0.999594	MYD88	ENSG00000172936
eqtl-a-ENSG00000127580	Inverse variance weighted (fixed effects)	3	0.001999	0.000859	0.019958	1.002001	1.000315	1.003689	WDR24	ENSG00000127580
eqtl-a-ENSG00000166851	Inverse variance weighted (fixed effects)	4	0.002397	0.000987	0.015194	1.0024	1.000462	1.004341	PLK1	ENSG00000166851
eqtl-a-ENSG00000166908	Inverse variance weighted (fixed effects)	5	0.002635	0.00108	0.014648	1.002639	1.000519	1.004763	PIP4K2C	ENSG00000166908
eqtl-a-ENSG00000149212	Inverse variance weighted (fixed effects)	10	-0.00122	0.000483	0.011515	0.99878	0.997834	0.999726	SESN3	ENSG00000149212
eqtl-a-ENSG00000152409	Inverse variance weighted (fixed effects)	9	0.001	0.000389	0.010207	1.001001	1.000237	1.001765	JMY	ENSG00000152409
eqtl-a-ENSG00000168329	Inverse variance weighted (fixed effects)	11	0.001603	0.000621	0.009879	1.001604	1.000385	1.002825	CX3CR1	ENSG00000168329
eqtl-a-ENSG00000185043	Inverse variance weighted (fixed effects)	12	-0.00064	0.000247	0.009634	0.999361	0.998877	0.999845	CIB1	ENSG00000185043
eqtl-a-ENSG00000239697	Inverse variance weighted (fixed effects)	8	-0.00097	0.000372	0.009179	0.999031	0.998302	0.99976	TNFSF12	ENSG00000239697
eqtl-a-ENSG00000100330	Inverse variance weighted (fixed effects)	9	-0.00084	0.000317	0.008026	0.999159	0.998538	0.999781	MTMR3	ENSG00000100330
eqtl-a-ENSG00000204209	Inverse variance weighted (fixed effects)	5	0.002393	0.000884	0.006765	1.002396	1.000661	1.004134	DAXX	ENSG00000204209
eqtl-a-ENSG00000110395	Inverse variance weighted (fixed effects)	5	-0.00203	0.000734	0.005693	0.997973	0.996538	0.999409	CBL	ENSG00000110395
eqtl-a-ENSG00000176720	Inverse variance weighted (fixed effects)	7	-0.00193	0.000698	0.00565	0.99807	0.996706	0.999437	BOK	ENSG00000176720
eqtl-a-ENSG00000197635	Inverse variance weighted (fixed effects)	6	-0.00347	0.001228	0.004746	0.996538	0.994142	0.99894	DPP4	ENSG00000197635
eqtl-a-ENSG00000240972	Inverse variance weighted (fixed effects)	3	0.003252	0.001107	0.003325	1.003257	1.001081	1.005437	MIF	ENSG00000240972
eqtl-a-ENSG00000121879	Inverse variance weighted (fixed effects)	4	-0.00371	0.001238	0.002709	0.996296	0.993882	0.998715	PIK3CA	ENSG00000121879
eqtl-a-ENSG00000181649	Inverse variance weighted (fixed effects)	3	-0.00383	0.001258	0.002326	0.996178	0.993726	0.998636	PHLDA2	ENSG00000181649
eqtl-a-ENSG00000129354	Inverse variance weighted (fixed effects)	7	-0.00188	0.000616	0.002258	0.99812	0.996915	0.999326	AP1M2	ENSG00000129354
eqtl-a-ENSG00000010810	Inverse variance weighted (fixed effects)	4	0.004596	0.001497	0.002138	1.004606	1.001663	1.007558	FYN	ENSG00000010810
eqtl-a-ENSG00000147649	Inverse variance weighted (fixed effects)	4	-0.00486	0.001562	0.001873	0.995156	0.992114	0.998206	MTDH	ENSG00000147649
eqtl-a-ENSG00000039068	Inverse variance weighted (fixed effects)	13	0.001779	0.000548	0.001177	1.00178	1.000704	1.002857	CDH1	ENSG00000039068
eqtl-a-ENSG00000137713	Inverse variance weighted (fixed effects)	7	0.001369	0.000415	0.000958	1.00137	1.000557	1.002184	PPP2R1B	ENSG00000137713
eqtl-a-ENSG00000103657	Inverse variance weighted (fixed effects)	5	-0.003	0.000893	0.000792	0.997008	0.995264	0.998754	HERC1	ENSG00000103657
eqtl-a-ENSG00000188906	Inverse variance weighted (fixed effects)	13	0.002033	0.000598	0.000669	1.002035	1.000862	1.003209	LRRK2	ENSG00000188906
eqtl-a-ENSG00000101421	Inverse variance weighted (fixed effects)	20	0.001154	0.000332	0.000507	1.001155	1.000504	1.001807	CHMP4B	ENSG00000101421
eqtl-a-ENSG00000186010	Inverse variance weighted (fixed effects)	5	0.003042	0.000849	0.000337	1.003047	1.00138	1.004717	NDUFA13	ENSG00000186010
eqtl-a-ENSG00000213145	Inverse variance weighted (fixed effects)	5	0.00625	0.001672	0.000185	1.00627	1.002978	1.009572	CRIP1	ENSG00000213145
eqtl-a-ENSG00000112425	Inverse variance weighted (fixed effects)	14	-0.00122	0.000326	0.000183	0.998781	0.998142	0.999419	EPM2A	ENSG00000112425
eqtl-a-ENSG00000183765	Inverse variance weighted (fixed effects)	7	0.005331	0.001361	8.96E-05	1.005345	1.002667	1.008031	CHEK2	ENSG00000183765
eqtl-a-ENSG00000143514	Inverse variance weighted (fixed effects)	4	-0.00348	0.000887	8.53E-05	0.996522	0.994791	0.998255	TP53BP2	ENSG00000143514
eqtl-a-ENSG00000160803	Inverse variance weighted (fixed effects)	3	0.007662	0.001581	1.26E-06	1.007691	1.004573	1.010819	UBQLN4	ENSG00000160803
eqtl-a-ENSG00000064547	Inverse variance weighted (fixed effects)	17	-0.00139	0.000267	1.88E-07	0.998607	0.998084	0.999131	LPAR2	ENSG00000064547
eqtl-a-ENSG00000132676	Inverse variance weighted (fixed effects)	10	-0.00358	0.000458	5.21E-15	0.996423	0.995529	0.997318	DAP3	ENSG00000132676

### 3.3 Recognition of PIK3CA, SESN3, ANXA5, MYD88, DPP4, DAXX, and CRIP1 as prognostic genes

**Figure 2:**
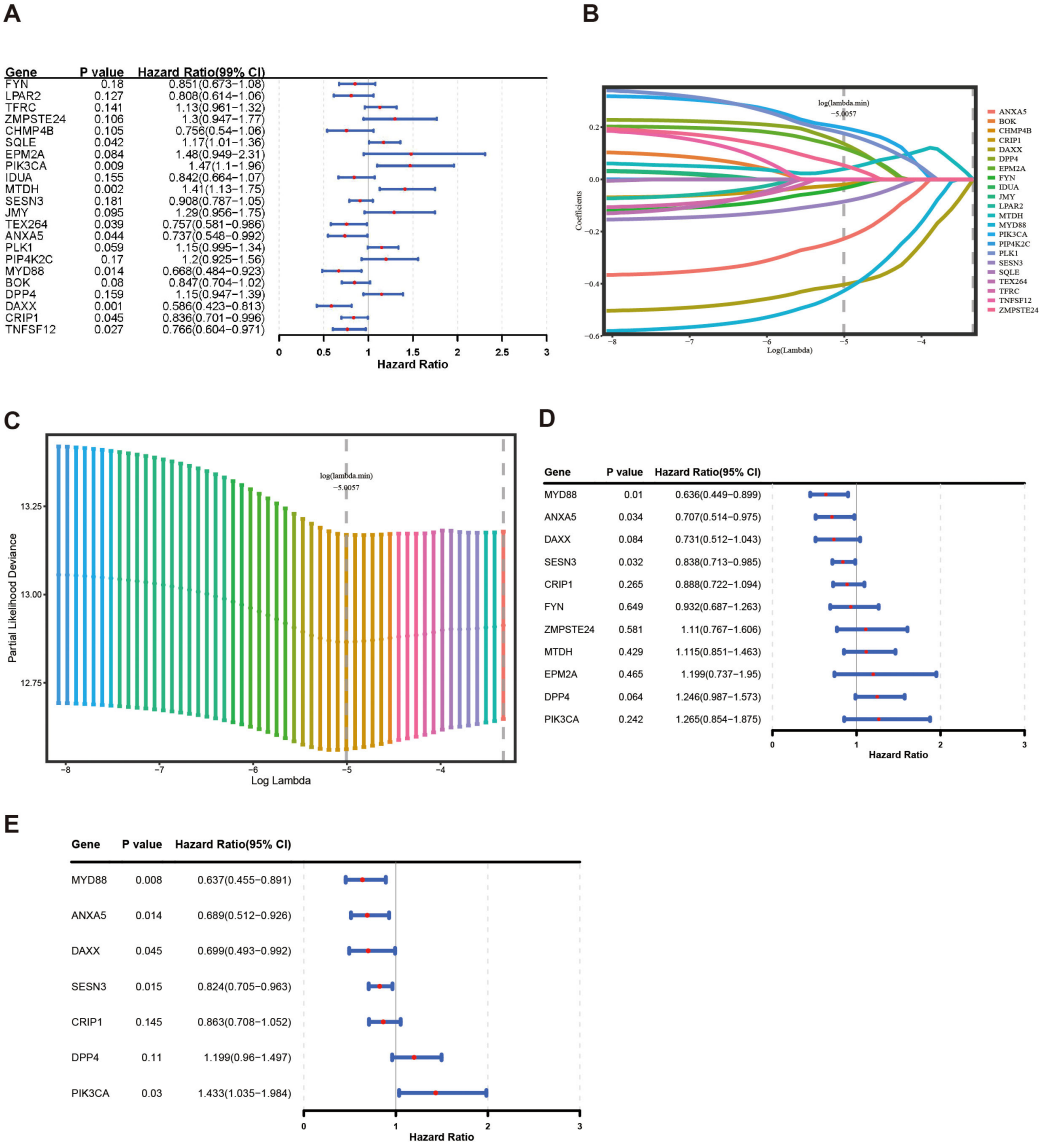
Construction of a risk model by Cox regression and LASSO analysis in the training group. **(A)** The forest plot for univariate Cox analysis. **(B, C)** LASSO regression was used to determine the optimal outcome λ values. **(D, E)** The forest plot for multivariate COX analysis and stepwise regression analysis.

After 54 key genes were obtained, 22 survival-associated genes were identified by univariate Cox regression analysis (*p* < 0.2) and PH assumption test (*p* > 0.05) (**Figure 2A**, **Table 2**). Then, these 22 genes were incorporated into LASSO analysis (lambda.min = 0.006699639), which yielded a total of 12 significant genes (FYN, ZMPSTE24, EPM2A, PIK3CA, MTDH, SESN3, ANXA5, PLK1, MYD88, DPP4, DAXX, and CRIP1) with a log(lambda.min ≠ 0 (**Figures 2B, C**). Finally, the PH assumption test excluded PLK1, followed by multivariate Cox analysis. The model was then screened and validated by stepwise regression analysis, resulting in seven prognostic genes (PIK3CA, SESN3, ANXA5, MYD88, DPP4, DAXX, and CRIP1) with *p* < 0.05 (**Figures 2D, E**; **Table 3**).

**Table 2:** Results of univariate Cox regression analysis and PH assumption test.

Gene	P_value
BAD	0.455852
CASP10	0.552484
FYN	0.917774
CDH1	0.096991
LPAR2	0.289792
ERBB3	0.149579
MYLK	0.65049
TFRC	0.312696
ZMPSTE24	0.417722
NLRP1	0.775306
MTMR3	0.056764
CHMP4B	0.175083
ARHGEF7	0.835618
HERC1	0.074423
SQLE	0.956928
CBL	0.28711
EPM2A	0.424358
PIK3CA	0.220091
IDUA	0.618828
WDR24	0.940641
AP1M2	0.940262
DAP3	0.196975
PPP2R1B	0.53946
UACA	0.303869
TP53BP2	0.45382
PARP1	0.028492
MTDH	0.793298
SESN3	0.593774
JMY	0.345596
BMP6	0.997127
UBQLN4	0.704015
TEX264	0.42449
ANXA5	0.362315
TP53INP1	0.094076
PLK1	0.058812
PIP4K2C	0.300765
CX3CR1	0.941204
PRELID1	0.127376
CXCL8	0.219645
SYNPO2	0.009942
MYD88	0.712093
SFN	0.026588
BOK	0.748919
PHLDA2	0.002169
CHEK2	0.458157
CIB1	0.965798
NDUFA13	0.199574
LRRK2	0.325526
ATP2A1	0.260043
DPP4	0.258957
DAXX	0.486589
CRIP1	0.828803
TNFSF12	0.820692
MIF	0.157154

**Table 3:** Results of stepwise regression analysis.

Gene	P_value
FYN	0.546473
ZMPSTE24	0.271245
EPM2A	0.635281
PIK3CA	0.34695
MTDH	0.838477
SESN3	0.57122
ANXA5	0.108482
PLK1	0.024894
MYD88	0.657923
DPP4	0.285684
DAXX	0.258309
CRIP1	0.833499

### 3.4 Development of a risk model with high accuracy

**Figure 3:**
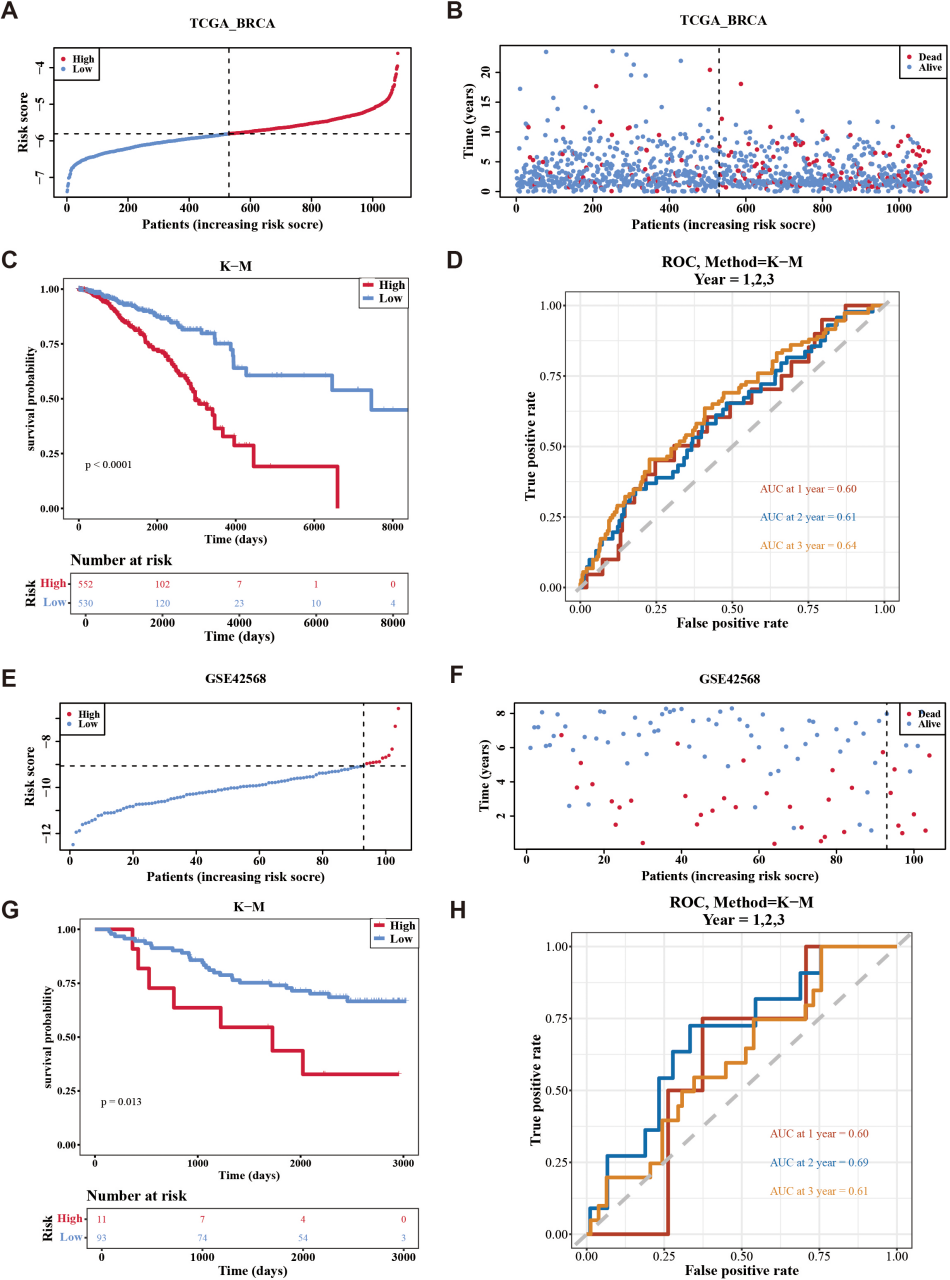
Evaluation the accuracy of the risk model in training set and validation set. **(A, B)** Distribution of the risk score and survival status between low-/high-risk groups in the training set. **(C)** Kaplan-Meier survival analysis between low-/high-risk groups in the training set. **(D)** Time-dependent receiver operating characteristic curve analysis of the training set. **(E, F)** Distribution of the risk score and survival status between low-/high-risk groups in GSE42568 data set. **(G)** Kaplan-Meier survival analysis between low- and high-risk subgroups of patients in GSE42568 data set. **(H)** Time-dependent receiver operating characteristic curve analysis of the GSE42568 data set.

The risk model was formulated using the expression intensity and risk coefficients of seven prognostic genes. Constructed risk model was as follows: RiskScore = PIK3CA × 0.36 + SESN3 × (–0.19) + ANXA5 × (–0.37) + MYD88 × –0.45) + DPP4 × 0.18 + DAXX × (–0.36) + CRIP1 × (–0.15). According to this model, patients in TCGA-BC were classified into high-risk (n = 552) and low-risk (n = 530) groups using an optimal cutoff value (–5.80621) for risk score. The data indicated that the number of deaths increased as the risk score increased (**Figures 3A, B**). Individuals in the high-risk group experienced significantly lower survival rates (*p* < 0.0001) (**Figure 3C**). ROC analysis suggested that the risk model exhibited good predictive capacity, with Area Under the Curves (AUC) for 1 (0.60), 2 (0.61), and 3 (0.64) years in TCGA-BC (**Figure 3D**).

Furthermore, patients were divided into high-risk (n = 11) and low-risk (n = 93) groups using an optimal cutoff value of (–9.06375) for risk score in GSE42568. Similarly, the number of deaths increased as the risk score in the sample increased (**Figures 3E, F**), with individuals in the high-risk cohort experiencing decreased survival rates (*p* = 0.013) (**Figure 3G**). Validation of the risk model in the GSE42568 dataset confirmed its predictive accuracy, as evidenced by AUC. The values were 0.60, 0.69, and 0.61 for 1, 2, and 3 years, respectively, underscoring the model’s consistent prognostic strength (**Figure 3H**). These outcomes affirmed the robustness of the risk model in evaluating the prognostic risk of patients with BC.

In addition, the Kaplan-Meier curves between the high and low expression groups of prognostic genes showed that the survival rate was significantly lower when the expression levels of DPP4 and PIK3CA were high (*p* < 0.05), while the survival rate was significantly lower when the expression levels of the remaining five prognostic genes were low (*p* < 0.05). In general, this further illustrated the prognostic value of prognostic genes for breast cancer patients.

### 3.5 Risk score, age, and stage were independent prognostic factors in TCGA-BC

**Figure 4:**
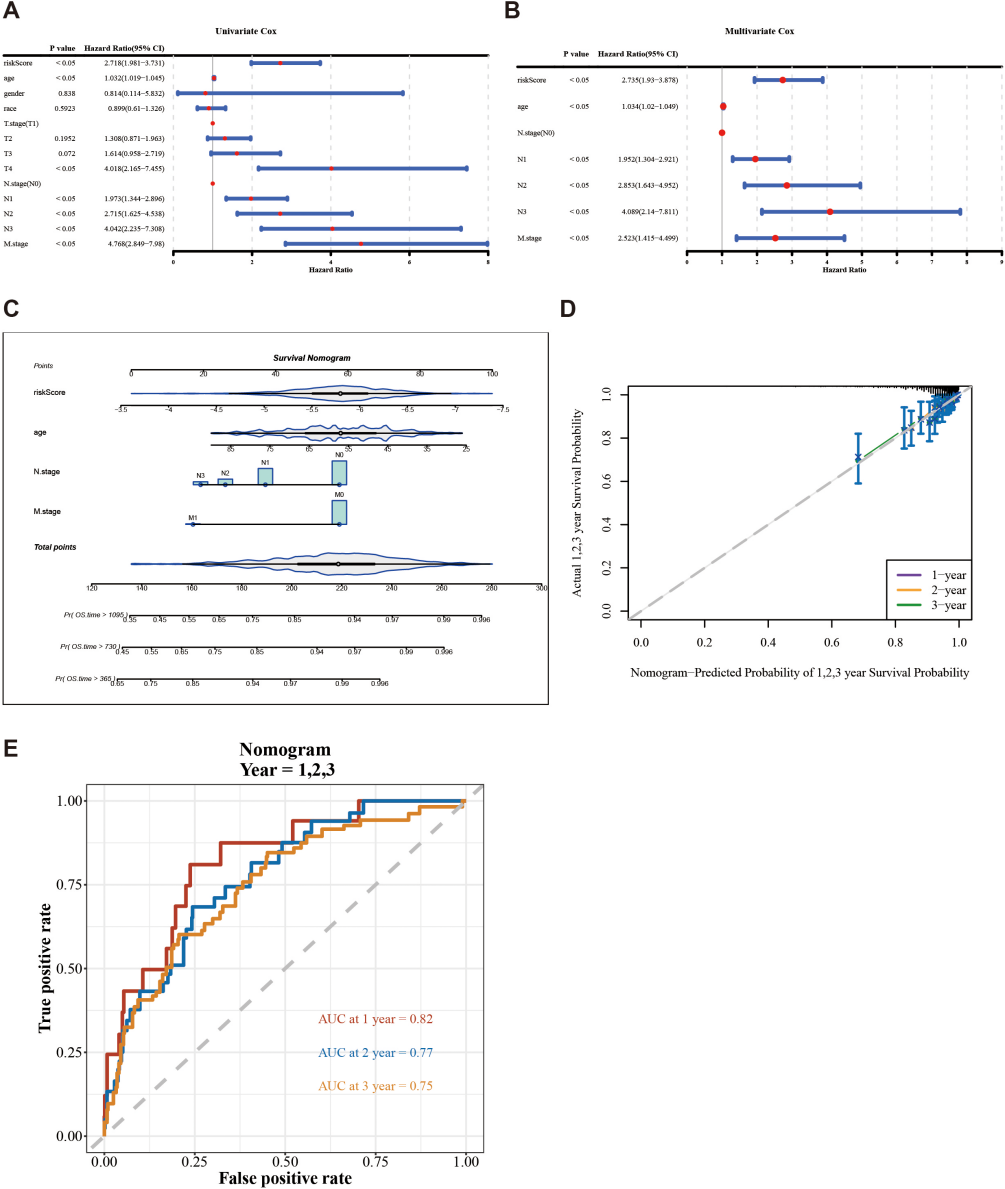
Construction and evaluation of a nomogram based on independent prognostic indicators of BC. **(A, B)** Univariate and multivariate Cox regression analyses were used to identify the independent prognostic indicators for BC on the clinical characteristics and the risk score. **(C)** Construction of a nomogram in conjunction with variables that independently predict BC prognosis. **(D)** Calibration plot of the nomogram. **(E)** AUC for predicting 1-, 2-, and 3-year overall survival for comparing the accuracy of various prognostic characteristics.

The risk score, age, N.stage, and M.stage were recognized as independent prognostic factors of TCGA-BC (*p* < 0.05) (**Figures 4A, B**). The nomogram model constructed by independent prognostic factors indicated that the risk of breast cancer was influenced by independent prognostic factors (**Figure 4C**). The resulting calibration curve reflected the nomogram’s high predictive precision for patient outcomes at 1, 2, and 3 years’ intervals (**Figure 4D**). The ROC analysis of 1 (0.82), 2 (0.77), and 3 years (0.75) in TCGA-BC (**Figure 4E**) indicates that the predictive performance of the nomogram plot is good.

**Figure 5:**
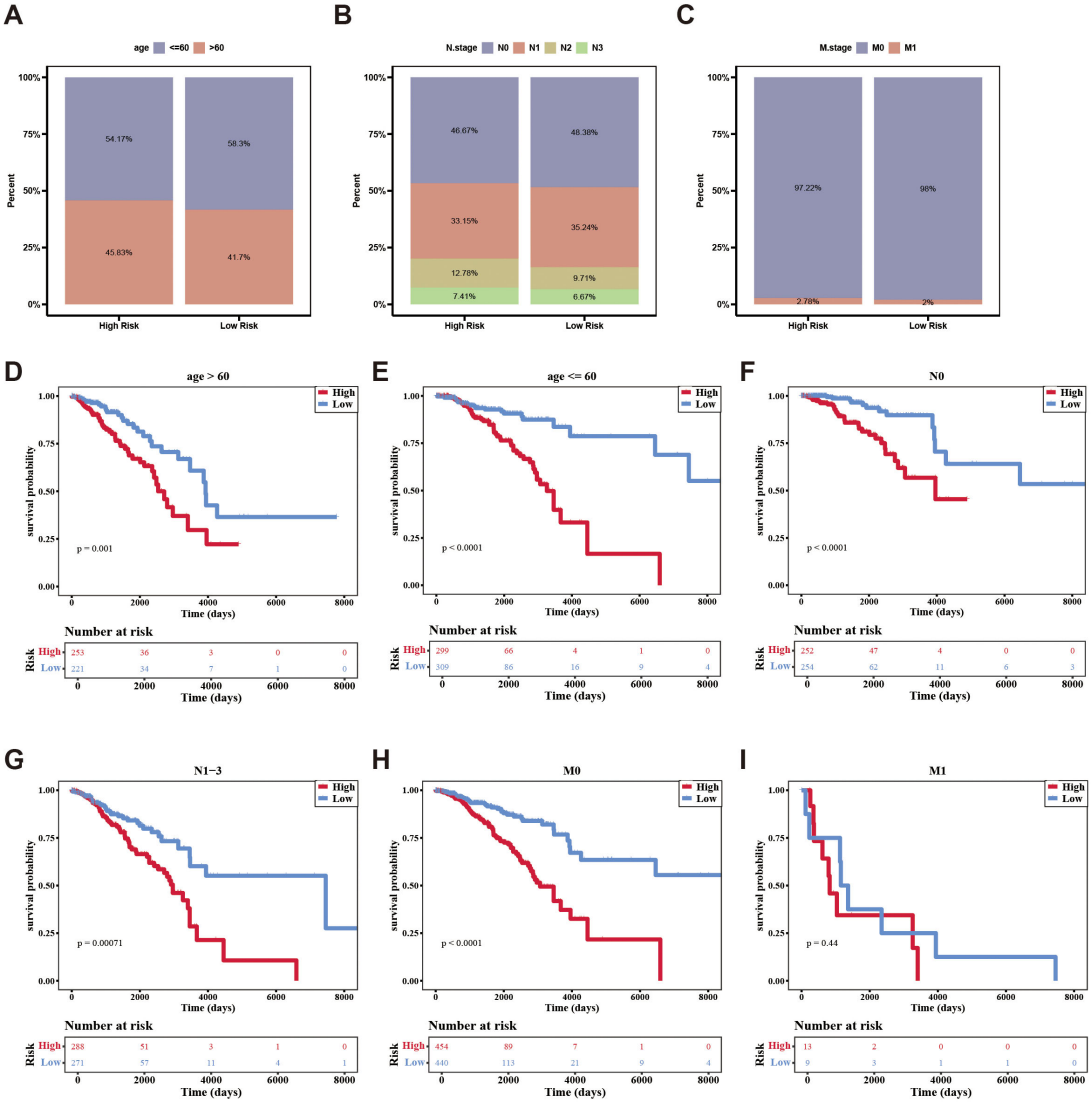
The distribution of different clinical characteristics between low-/high-risk BC patients in independent prognostic factors. **(A–C)** Histogram shows the distribution of different clinical characteristics in high-risk and low-risk groups. **(D–I)** Kaplan-Meier survival analysis was used to compare the survival differences low-/high-risk groups in different clinical characteristics subtypes.

Additionally, the survival rates of the two risk groups were analyzed among different clinical subgroups. Patients were more prevalent in the high-risk group within age (> 60), N.stage (N2, N3), and M.stage (M1). Conversely, patients were more frequently found in the low-risk group within age (≦60), N.stage (N0, N1), and M.stage (M0) (**Figures 5A–C**). Survival differences between the two risk groups across various clinical subtypes indicated that patients in the high-risk group exhibited reduced survival rates (age, N.stage, and M.stage [M0]) (**Figures 5D–I**).

In conclusion, the aforementioned results not only demonstrated the impact of clinical characteristics on the survival of breast cancer patients but also indicated that certain independent prognostic features were associated with the survival risk of these patients.

### 3.6 Biological pathway and mutation genes analysis of risk groups in BC

**Figure 6:**
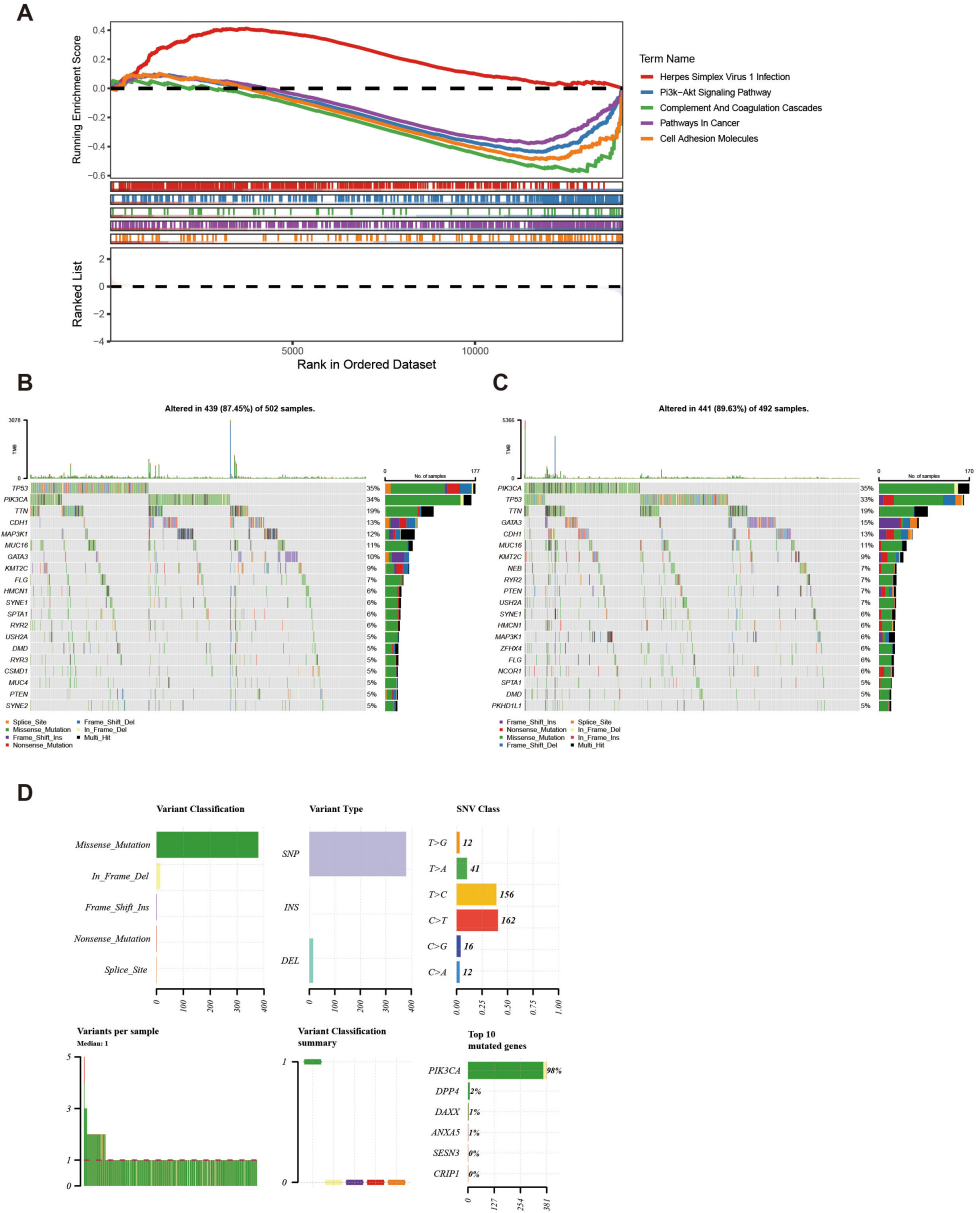
KEGG and genomic variant analysis between low-/high-risk BC patients. **(A)** KEGG analysis of the high- and low-risk groups. **(B, C)** Gene mutation frequency analysis in the high- **(B)** and low-risk groups **(C)**. **(D)** Summary of the mutational analysis including variant classification, variant type, SNV class, variants per sample, variant classification summary, and the top 10 mutated genes.

Enrichment analysis of all genes in TCGA-BC resulted in a total of 30 pathways, such as herpes simplex virus 1 infection (**Figure 6A**, **Supplementary Table 9**). This pathway might be associated with the immune evasion of breast cancer.

Furthermore, within the high-risk group, TP53, PIK3CA, and TTN genes resulted in high mutation rates, reaching 35%, 34%, and 19% respectively (**Figure 6B**). Conversely, in the low-risk group, PIK3CA, TP53, and TTN genes indicated a high mutation rate, achieving 35%, 33%, and 19% respectively (**Figure 6C**). Further analysis of the mutational panorama of prognostic genes revealed that prognostic genes comprised the highest number of missense mutations and occupied the most SNP mutation types. Additionally, the highest percentage of single-nucleotide variants was found for T > C and C > T, while the PIK3CA mutated genes occurred at the highest frequency (**Figure 6D**). The aforementioned results indicated that the TP53, PIK3CA, and TTN genes exhibited high mutation rates with similar frequencies in both two groups. Meanwhile, missense mutations and SNP types were the predominant mutation patterns among prognostic genes, with T > C/C > T single-nucleotide variants and PIK3CA gene mutations being the most common. These findings provided a basis for revealing the molecular mutation characteristics associated with breast cancer risk stratification.

### 3.7 The changes in immune cells and the differences in drug sensitivity among different risk groups

**Figure 7:**
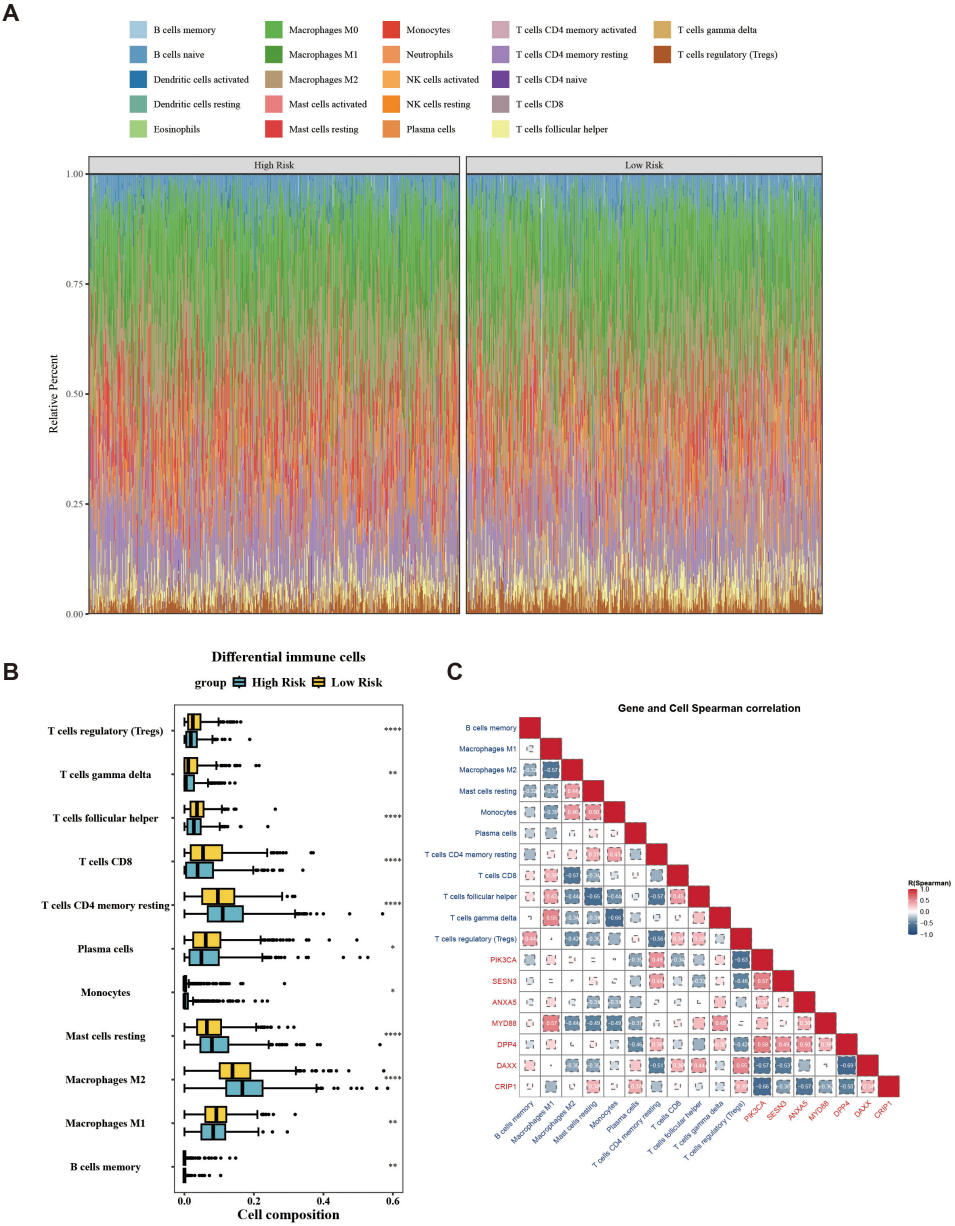
Landscape of tumor-infiltrating immune cells abundance between low-/high-risk BC patients. **(A)** Stacked graph of the proportions of various types of immune cells in the high- and low-risk groups. **(B)** Differences in the infiltration of immune cells between the high- and low-risk groups. **(C)** Correlation between differential immune cells and prognostic genes. ^*^p < 0.05; ^**^p < 0.01; ^****^p < 0.0001.

In the immune microenvironment, the proportion of abundance of 22 immune cells in two risk groups is illustrated in **Figure 7A**. The immune cells with notable distinctions were analyzed in two risk groups (*p* < 0.05). This analysis revealed 11 immune cells that were notably different between the two groups, such as macrophages M2, CD8 T cells, and regulatory T cells (Tregs) (**Figure 7B**). The relevance between prognostic genes and differential immune cells indicated that PIK3CA was most counterintuitively linked to Tregs (cor = –0.63). Conversely, MYD88 demonstrated a positive correlation with M1 Macrophages (cor = 0.57). The correlation between differential immune cells displays that gamma delta T cells exhibited a negative correlation with monocytes (cor = –0.66), while gamma delta T cells displayed a positive correlation with macrophages M1 (cor = 0.55). The correlation between prognostic genes indicated a strong inverse correlation between DPP4 and DAXX (cor = –0.69), while PIK3CA exhibited a significant positive correlation with DPP4 (cor = 0.58) (**Figure 7C**). Overall, the state of immune cells might affect the risk of breast cancer, further highlighting the significance of prognostic genes.

**Figure 8:**
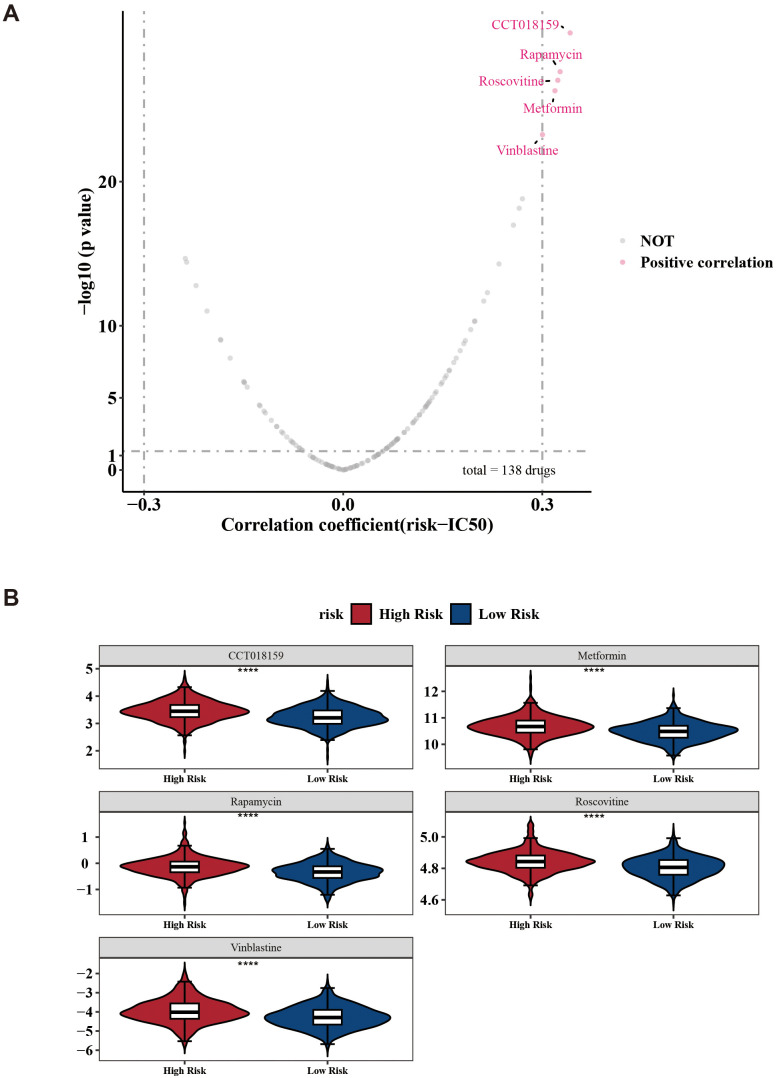
Drug sensitivity prediction between low-/high-risk BC patients. **(A)** Volcano plots show the correlation between IC50 and risk score in high- and low-risk groups. **(B)** Boxplots indicating significant differences in estimated IC50 values of 5 potential drugs. ^****^p < 0.0001.

Next, the analysis revealed detailed information for common chemotherapeutic drugs, including 138 drugs, with CCT018159, rapamycin, vinblastine, metformin, and roscovitine being notably different between the two risk groups (**Figure 8A**). Among these, the IC_50_ of CCT018159, rapamycin, vinblastine, metformin, and roscovitine in the high-risk group were higher in the high-risk group (|cor| > 0.3 and *p* < 0.05) (**Figure 8B**). Patients in the low-risk groups of CCT018159, rapamycin, vinblastine, metformin, and roscovitine were more sensitive. These drugs might be suitable for treating patients in the low-risk group. In conclusion, the aforementioned results provided a new theoretical basis for the pharmacotherapy of different risk groups.

### 3.8 Validation of the expression and functional roles of MYD88, ANXA5, DAXX, SESN3, CRIP1, DPP4, and PIK3CA

**Figure 9:**
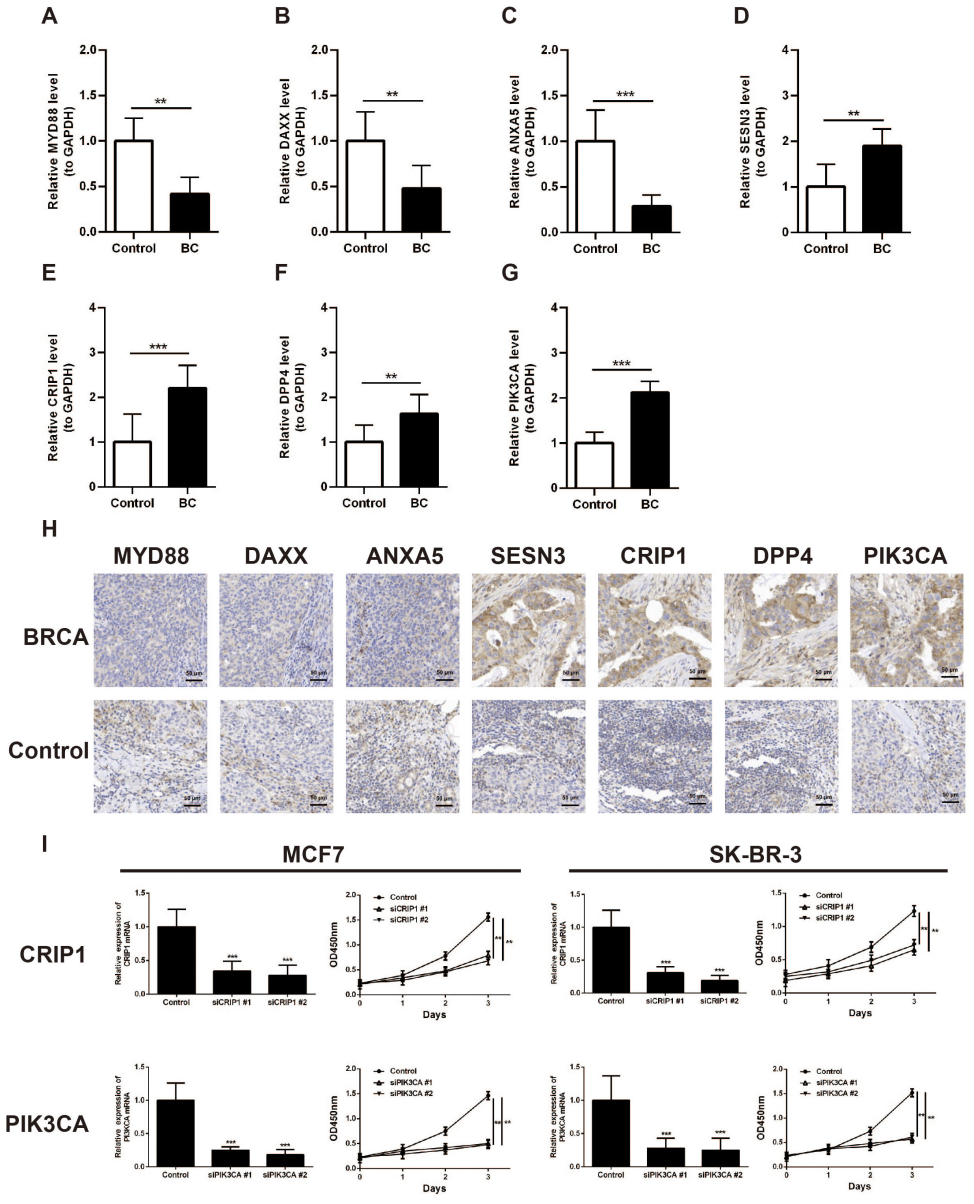
Validation of the expression of MYD88, ANXA5, DAXX, SESN3, CRIP1, DPP4, and PIK3CA in breast cancer clinical samples. **(A)** Analysis of MYD88 expression in BC tissues and paired control tissues by RT-qPCR. **(B)** Analysis of ANXA5 expression in BC tissues and paired control tissues by RT-qPCR. **(C)** Analysis of DAXX expression in BC tissues and paired control tissues by RT-qPCR. **(D)** Analysis of SESN3 expression in BC tissues and paired control tissues by RT-qPCR. **(E)** Analysis of CRIP1 expression in BC tissues and paired control tissues by RT-qPCR. **(F)** Analysis of DPP4 expression in BC tissues and paired control tissues by RT-qPCR. **(G)** Analysis of PIK3CA expression in BC tissues and paired control tissues by RT-qPCR. **(H)** Immunohistochemistry analysis of MYD88, ANXA5, DAXX, SESN3, CRIP1, DPP4, and PIK3CA expression in BRCA tissues and paired control tissues. **(I)** CCK8 assay was carried out to detect the proliferation of control and CRIP1 or PIK3CA knockdown BRCA cell lines. ^**^p < 0.01; ^***^p < 0.001.

The expression of seven prognostic genes was estimated by RT-qPCR and IHC in 32 matched pairs of breast cancer and adjacent paracancerous tissues (**Table 4**). The expression levels of MYD88, ANXA5, DAXX were significantly upregulated in the control samples compared to BC samples (*p* < 0.05) (**Figures 9A–C**). However, the expression levels of SESN3, CRIP1, DPP4, and PIK3CA were significantly upregulated in the BC samples compared to control samples (*p* < 0.05) (**Figures 9D–G**). Immunohistochemistry was utilized to evaluate the expression levels of these prognostic genes within BRCA tissues and paired control tissues. The results revealed that the expression levels of SESN3, CRIP1, DPP4, and PIK3CA was higher than that of MYD88, ANXA5, DAXX (**Figure 9H**). Additionally, the CCK8 assay results indicated that PIK3CA and CRIP1 significantly promotes the proliferation of BRCA cells (**Figure 9I**). The above results have enhanced the reliability of the bioinformatics analysis results.

**Table 4:** Clinical and pathological information for breast cancer patients.

Sample	Age	Pathology diagnosis	Stage	PR	ER	HER2	Subtypes
1	54	Ductal infiltrating carcinoma	IIB	+++	++	–	Luminal A
2	36	Infiltrating lobular carcinoma	IIA	+++	++	–	Luminal A
3	42	Ductal infiltrating carcinoma	IIB	++	–	–	Luminal B
4	53	Ductal infiltrating carcinoma	IIIA	–	–	+++	HER2 positive
5	57	Ductal infiltrating carcinoma	IIB	+++	+++	–	Luminal A
6	56	Infiltrating lobular carcinoma	IIB	–	–	–	TNBC
7	48	Infiltrating lobular carcinoma	IIB	++	–	+++	HER2 positive
8	49	Ductal infiltrating carcinoma	IIB	+++	++	–	Luminal A
9	61	Ductal infiltrating carcinoma	IIA	–	–	–	TNBC
10	71	Ductal infiltrating carcinoma	IIA	+++	+++	–	Luminal A
11	51	Ductal infiltrating carcinoma	IIA	++	+	–	Luminal B
12	50	Ductal infiltrating carcinoma	IIB	–	–	+++	HER2 positive
13	40	Ductal infiltrating carcinoma	IIB	+++	++	–	Luminal A
14	57	Infiltrating lobular carcinoma	IIA	+++	+++	–	Luminal A
15	61	Ductal infiltrating carcinoma	IIA	–	–	–	TNBC
16	35	Ductal infiltrating carcinoma	IIB	+	–	+++	HER2 positive
17	57	Infiltrating lobular carcinoma	IIA	+++	++	–	Luminal A
18	85	Infiltrating carcinoma	IIIB	+++	+++	–	Luminal A
19	79	Infiltrating carcinoma	IIB	–	–	+++	HER2 positive
20	58	Ductal infiltrating carcinoma	IIIB	++	+	–	Luminal B
21	63	Ductal infiltrating carcinoma	IIB	+++	++	–	Luminal A
22	50	Infiltrating carcinoma	IIA	–	–	–	TNBC
23	49	Infiltrating carcinoma	IIIA	+++	+++	–	Luminal A
24	53	Ductal infiltrating carcinoma	IIA	+	–	+++	HER2 positive
25	46	Ductal infiltrating carcinoma	IIA	+++	++	–	Luminal A
26	46	Ductal infiltrating carcinoma	IIIB	–	–	++	HER2 positive
27	41	Infiltrating carcinoma	IIB	+++	+++	–	Luminal A
28	42	Ductal infiltrating carcinoma	IIIB	–	–	–	TNBC
29	51	Ductal infiltrating carcinoma	IIIB	++	+	–	Luminal B
30	44	Ductal infiltrating carcinoma	IIA	+++	++	–	Luminal A
31	44	Ductal infiltrating carcinoma	IIB	+++	+++	–	Luminal A
32	43	Ductal infiltrating carcinoma	IIIC	–	–	–	TNBC

## 4 Discussion

Globally, BC is the most commonly diagnosed cancer and the leading cause of cancer-related deaths among women (44). The selection of treatment regimens for BC, including surgical resection, radiotherapy, endocrine therapy, targeted therapy, and systemic chemotherapy, is determined by its molecular and histological characteristics (45). However, certain breast cancer subtypes are associated with limited therapeutic options and poor survival outcomes (46). Immunotherapy, a promising approach for cancer treatment, has emerged as a central focus in oncology, utilizing immune checkpoint inhibitors as a key therapeutic strategy (47). Therefore, prognostic biomarkers are imperative to assess patient outcomes and guide therapeutic strategies. Evidence indicates that m6A can regulate the expression and function of programmed cell death processes, including apoptosis, pyroptosis, ferroptosis, autophagy, and necroptosis (48). Currently, this approach is being applied to provide potential therapeutic targets, anticancer agents, or combination therapies for cancer treatment (49). However, existing studies have primarily focused on the individual roles of either m6A (50) or PCD (51) in breast cancer, while their combined impact remains unreported.

In this study, we identified seven potential prognostic genes for BC: PIK3CA, SESN3, ANXA5, MYD88, DPP4, DAXX, and CRIP1. PIK3CA encodes the p110α catalytic subunit of PI3K, an enzyme critical for intracellular signal transduction. Mutations in the PIK3CA gene are associated with the development of multiple cancer types (52). In colorectal cancer, the m6A modification pattern correlates with tumor mutational burden and reveals a link through which m6A, by associating with PIK3CA mutations, participates in regulating the cancer immune microenvironment and therapeutic response (53). Multiple studies have shown that PIK3CA is implicated in the pathogenesis and progression of breast cancer (54, 55). SESN3 is a member of the Sestrin family. Sestrins are stress-inducible proteins that may play significant roles in the pathogenesis of multiple diseases, including cancer, metabolic disorders, and neurodegenerative diseases (56). Our research reveals a novel role for the SESN3 gene in influencing breast cancer progression via m6A or PCD, which paves the way for in-depth future studies. Furthermore, ANXA5 is a calcium-dependent phospholipid-binding protein belonging to the annexin family. It plays critical roles in maintaining membrane stability, regulating endocytosis, and modulating cellular proliferation, differentiation, apoptosis, as well as inflammation and thrombosis processes (57). Previous studies have shown that ANXA5 can influence tumor progression through m6A (58) or PCD (57), but its role in breast cancer remains to be further investigated. MYD88 is an adaptor protein that plays a pivotal role in immune responses. It is closely associated with multiple diseases, including cancers, infectious diseases, and autoimmune disorders (59). However, it remains unclear whether MYD88 influences the progression of breast cancer through m6A or PCD. Additionally, DPP4 is a serine exopeptidase, and its inhibitors represent a novel therapeutic option for type 2 diabetes mellitus. These inhibitors may also have potential therapeutic applications for other diseases (60). Wang et al. found that IGF2BP2 promotes lymphatic metastasis via stabilizing DPP4 in an m6A-dependent manner in papillary thyroid carcinoma (61). And whether DPP4 influences breast cancer progression through m6A requires further investigation. DAXX is a multifunctional protein that plays a role in regulating gene expression, DNA repair, cell cycle control, and tumor suppression (62). In triple negative breast cancer, DAXX protein can trigger Caspase-3 which prompts apoptosis by cleaving PARP-1 protein (63). Conversely, CRIP1 is a cysteine-rich protein that exhibits aberrant expression in multiple malignancies, including breast cancer, cervical cancer, and pancreatic carcinoma. It contributes significantly to tumor cell proliferation, migration, and invasion processes (64). Our study first identified CRIP1 might participate breast cancer progression through m6A manner.

Then, we developed a risk prognostic model based on expression levels of PIK3CA, SESN3, ANXA5, MYD88, DPP4, DAXX, and CRIP1 to predict clinical outcomes in patients with breast cancer. Performance validation demonstrated high predictive accuracy for prognostic model, further indicating the prognostic value of prognostic genes. Additionally, GSEA, mutation status, immune infiltration profiling, and drug sensitivity assays were employed to characterize enriched biological pathways and assess differential immune cell infiltration and therapeutic response between high- and low-risk BC cohorts. In gene set enrichment analysis, we found the herpes simplex virus 1 infection signal pathway exhibits higher activity, which may facilitate immune evasion in breast cancer cells potentially by modulating their immunogenicity or interfering with the anti-tumor immune response. M1 macrophages were identified as one of the differentially abundant immune cells. It has been reported that overexpression of certain genes can suppress the progression of breast cancer subtypes by promoting M1 macrophage polarization. In line with the immune infiltration profiling results, MYD88 showed a significant positive correlation with M1 macrophages, and similarly, gamma delta T cells were also significantly positively correlated with M1 macrophages. Therefore, it is plausible that through its significant positive correlation with M1 macrophages, MYD88 may modulate the functional state of M1 macrophages, thereby influencing the progression of breast cancer. In drug sensitivity analysis, we found CCT018159, rapamycin, vinblastine, metformin, and roscovitine being notably different between the two risk groups, which provides a novel perspective for therapeutic strategies targeting high- and low-risk patient groups. Then, validation demonstrated that the integrated nomogram (incorporating independent predictors: Risk score, T stage, N stage, and M stage) achieved high predictive accuracy for 1-, 2-, and 3-year survival probabilities. Our clinical characteristic model can provide clinicians with a visual tool and evidence-based support for formulating personalized management strategies, such as intensified monitoring for high-risk groups and streamlined follow-up for low-risk cohorts. In conclusion, the poor outcomes and reduced survival in high-risk patients may be attributed to enhanced tumor immune evasion mechanisms and limited immunotherapy efficacy within this subgroup.

In summary, we identified seven prognostic genes related to m6A and PCD in breast cancer. Besides, we created a risk model and a nomogram model, revealing the prognostic value of prognostic genes and independent prognostic factors in breast cancer. Our findings open new avenues for research and establish a theoretical framework for treating BC and other malignancies with analogous pathogenesis. However, the findings derived from this study require further validation and confirmation through subsequent research.

Although we have developed a prognostic model grounded in the identified genes and have demonstrated their prognostic significance, a finding consistently corroborated by independent datasets, the considerable heterogeneity in molecular characteristics (such as hormone receptor status, HER2 expression, and mutational profiles) and clinical outcomes across various breast cancer subtypes prompts questions regarding the model’s generalizability to all subtypes. While gene expression differences were confirmed via qRT-PCR and IHC, protein-level validation using methods such as Western blotting has not yet been performed. Comprehensive functional experiments in breast cancer cell lines are still lacking, and the specific mechanisms by which these genes regulate tumor progression remain incompletely elucidated. Consequently, further research is necessary, including validation within larger, subtype-specific cohorts, to ascertain its applicability across different subtypes. Additionally, we intend to utilize cellular and animal models in future studies to further elucidate the functional mechanisms of these prognostic genes in breast cancer, with a particular focus on specific molecular subtypes.

## Data Availability

The datasets presented in this study can be found in online repositories. The names of the repository/repositories and accession number(s) can be found in the article/**Supplementary Material**.
