# Supplementary material for: A prognostic model for breast cancer survival based on PCD and m6A gene interactions
Source: Front Immunol. 2025 Nov 20;16:1711910. doi: 10.3389/fimmu.2025.1711910 (PMC12675348; doi:10.3389/fimmu.2025.1711910)
Supplement: Supplementary file 3 [file Table3.docx]

**Supplementary table 3**

| **Primers** | **Sequences** | |
| --- | --- | --- |
| MYD88-F | CGTTTCGATGCCTTCATCTGC | |
| MYD88-R | GTGGCCTTCTAGCCAACCTC | |
| ANXA5-F | TAATGCTCAGCGCCAGGAAA | |
| ANXA5-R | ACTTCTGCCTTAGCGGTTGG | |
| DAXX-F | CAGCAGAAGAGCGAAGACCA | |
| DAXX-R | GCTGGGTCTCGTAGTGGTTG | |
| SESN3-F | TTGCCAGTAGGCGATGCAA |  |
| SESN3-R | AGACTTGACTGGGGAAAGCG |  |
| CRIP1-F | GCAGCTGGTGTTTGTGAAGG |  |
| CRIP1-R | AGGAGGTGCTGATGATGGTG |  |
| DPP4-F | ATGCCAGGAGGAAGGAATCT |  |
| DPP4-R | TCCAGGACTCTCAGCCCTTT |  |
| PIK3CA-F | CCACGACCATCATCAGGTGAA |  |
| PIK3CA-R | CCTCACGGAGGCATTCTAAAGT |  |
| GAPDH-F | CGAAGGTGGAGTCAACGGATTT |  |
| GAPDH-R | ATGGGTGGAATCATATTGGAAC |  |
